# A self-guided Internet-delivered intervention for adults with ADHD: A feasibility study

**DOI:** 10.1016/j.invent.2021.100416

**Published:** 2021-06-15

**Authors:** Emilie S. Nordby, Robin M.F. Kenter, Astri J. Lundervold, Tine Nordgreen

**Affiliations:** a Division of Psychiatry, Haukeland University Hospital, Haukelandsbakken 15, 5009 Bergen, Norway; b Department of Clinical Psychology, Faculty of Psychology, University of Bergen, Christies gate 12, 5015 Bergen, Norway; c Department of Biological and Medical Psychology, Faculty of Psychology, University of Bergen, Jonas Lies vei 91, 5009 Bergen, Norway; d Department of Global Public Health and Primary Care, Faculty of Medicine, University of Bergen, Årstadveien 17, Block D, 5009 Bergen

**Keywords:** Attention Deficit Hyperactivity Disorder, ADHD, Adults, Internet-delivered interventions, Non-pharmacological treatment

## Abstract

**Background:**

Attention Deficit Hyperactivity Disorder (ADHD) is a prevalent neurodevelopmental disorder that tends to persist into adulthood. Many adults with ADHD seek non-pharmacological treatment in addition to pharmacological treatment. Still, there are few non-pharmacological treatment options available. The aim of the current study was to explore the feasibility of a self-guided Internet-delivered intervention for adults with ADHD.

**Methods:**

The study has an uncontrolled, within-group, pre-post design. Thirteen participants with an ADHD diagnosis were included and given access to the first three modules of a seven-module intervention. To explore the feasibility of the intervention, the adherence, credibility, and treatment satisfaction were examined. Preliminary efficacy of the intervention was examined through self-report measures of inattention, hyperactivity, depression, anxiety, stress, and quality of life.

**Results:**

In terms of adherence, seven participants (54%) completed all three intervention modules (*M* = 1.85, *SD* = 1.3). The participants reported both good credibility and treatment satisfaction with the intervention. The participants also reported challenges related to usability and technical issues. Although the clinical outcomes must be interpreted with caution due to the study design and the small sample size, a statistically significant reduction in severity of inattention was reported by the participants following the intervention (*p* = .006, *d* = 1.57). The reduction was non-significant for hyperactivity (*p* = .326, *d* = 0.33). The participants who completed all three modules in the intervention (*n* = 7) also reported a significant decrease in stress (*p* = .042, *d* = 0.67) and a significant increase in quality of life (*p* = .016, *d* = 0.99). No significant changes were found on measures of anxiety and depression.

**Conclusion:**

The adherence to the intervention was relatively low, but the participants who completed the study reported good credibility and satisfaction with the intervention. These results indicate that there is a need to improve the intervention to make it more engaging before conducting a randomized-controlled trial investigating the clinical effects of the full seven-module intervention.

## Highlights

• The study examined the feasibility of an Internet-delivered intervention for adults with ADHD.
• The participants reported good satisfaction and credibility.
• Seven out of thirteen included participants completed all intervention modules.
• The study provided information which enabled improvements of the intervention before conducting the main trial.

## 1 Introduction

Attention Deficit Hyperactivity Disorder (ADHD) is a neurodevelopmental disorder characterized by core symptoms of inattention and hyperactivity/impulsivity that impair everyday functioning across situations and time (American Psychiatric Association, 2013). ADHD tends to persist into adulthood with an estimated prevalence of 2–7% in adults (Faraone et al., 2006; Fayyad et al., 2017). Moreover, ADHD is associated with other comorbid symptoms and complaints, such as emotion dysregulation, low self-esteem, and self-harm (Allely, 2014; Harpin et al., 2016; Shaw et al., 2014). Together, these symptoms are known to affect function in several life domains, such as educational, occupational and interpersonal areas (Barkley, 2002; Murphy and Barkley, 1996). In addition, ADHD is associated with high rates of comorbidity, with the most common co-occurring disorders in adulthood being depression, anxiety, substance abuse disorders and personality disorders (Sobanski, 2006).

The first-line treatment for adults with ADHD is pharmacological treatment (National Institute for Health and Care Excellence, 2019). Pharmacological treatment is shown to reduce core symptoms of ADHD, but may also induce side-effects, such as insomnia and loss of appetite, or have an insufficient effect (Graham et al., 2011). Moreover, both responders and non-responders may still have residual impairments in other important life domains (Safren et al., 2010). Consequently, current treatment guidelines recommend a combination of non-pharmacological treatment and pharmacological treatment for adults with ADHD in order to meet their needs (National Institute for Health and Care Excellence, 2019).

In the past decade, an increasing amount of research has been dedicated to investigating the effectiveness of non-pharmacological interventions for adults with ADHD (Knouse and Safren, 2010; Solanto et al., 2018; Young et al., 2016). Most of these interventions are based on principles from Cognitive Behavioral Therapy (CBT) and studies have shown them to be effective in improving ADHD symptoms and other psychosocial problems (Knouse and Safren, 2010; Weiss et al., 2012). Other non-pharmacological treatment interventions, such as Dialectic Behavioral Therapy (DBT) and Goal Management Training (GMT) have also shown promising results for adults with ADHD (In de Braek et al., 2017; Philipsen et al., 2007). Given the many psychosocial domains that are affected by ADHD, combining therapeutic techniques from various psychotherapeutic frameworks should be beneficial. For instance, GMT mainly target inhibitory control, a primary deficit in individuals with ADHD (Barkley, 1997; Levine et al., 2011). However, GMT may fail to address other aspects of ADHD, such as emotion dysregulation, low self-esteem or self-acceptance, aspects that are strongly focused in DBT (Shaw et al., 2014; Swales and Heard, 2016), However, DBT does not emphasize the core aspects of executive functioning focused in GMT. As such, by integrating these psychotherapeutic frameworks, one should be able to provide an intervention addressing a broader range of symptoms and challenges related to ADHD. However, despite recommendations and the effort to develop and investigate non-pharmacological interventions for ADHD, these interventions are still unavailable to many adults with ADHD (Solberg et al., 2019). This is likely caused by several factors, including limited mental health care resources and lack of, or geographical distance to qualified clinicians and stigma (Kohn et al., 2004).

Interventions delivered over the Internet may help to increase access to effective non-pharmacological interventions for adults with ADHD. Internet-delivered interventions are found to be as effective as face-to-face interventions for several psychological disorders, such as depression (Andersson et al., 2016), anxiety disorders (Andersson, 2012), and insomnia (Ritterband et al., 2009). These interventions can both be guided, where the participant receives support from a therapist, and self-guided, where the participant goes through the intervention without therapist support. Self-guided interventions are expected to be a feasible option to deliver psychological interventions for adults with ADHD, as it may lower the threshold for seeking treatment and require less economic resources (Karyotaki et al., 2017).

To date, there are still few published studies examining Internet-delivered interventions for adults with ADHD. Pettersson et al. (2017) conducted a randomized-controlled trial of Internet-delivered CBT (iCBT), where 45 adults with ADHD were randomized into a self-guided iCBT intervention, an iCBT intervention with weekly group meetings, or a waiting-list control group (Pettersson et al., 2017). The reduction in self-rated ADHD symptoms was found to be significantly larger in the self-guided iCBT group than in the waiting list control group at the post-assessment, while the difference between the self-guided iCBT group and the iCBT group with weekly group meetings was statistically non-significant (Pettersson et al., 2017). With an aim to improve organizational skills with the support of smartphone applications, Moëll et al. (2015) conducted a randomized-controlled study of a CBT-inspired Internet-delivered intervention including 57 adults with ADHD (Moëll et al., 2015). The participants were randomized into an intervention group or a waiting list control group, and the intervention group showed a significantly stronger decline in inattentiveness following the intervention (Moëll et al., 2015). The abovementioned studies indicate that Internet-delivered interventions can provide a promising and feasible format to deliver psychological interventions for adults with ADHD. However, both these studies used a CBT framework, and there is still a lack of studies investigating interventions using other psychotherapeutic frameworks or combining techniques from various psychotherapeutic frameworks. As stated above, we believe that integrating elements from different psychotherapeutic frameworks is beneficial by targeting a broader range of challenges associated with ADHD.

The primary objective of the present study was to study the feasibility of a self-guided Internet-delivered intervention for adults with ADHD, by examining adherence, credibility, and treatment satisfaction of the first three modules of a full trial including a seven-module intervention. The current intervention had an integrative framework, including general principles and techniques from DBT, GMT and CBT. A secondary objective was to obtain preliminary information about the short-term clinical outcomes of the intervention on self-report scales assessing symptoms of inattention and hyperactivity. Considering that ADHD is a neurodevelopmental disorder, and that the diagnostic category in adults is defined by the persistence of these symptoms, we also included measures of stress, anxiety, depression, and quality of life, as an improvement in these areas are expected to improve overall functioning. The information from the feasibility study will be used to adapt the intervention before running the full trial.

## 2 Methods

### 2.1 Study design

The current study is a feasibility trial with an uncontrolled, within-group, pre-post design.

### 2.2 Sample

Eligible participants were adults with ADHD living in Western Norway. Participants were recruited from a local patient association who shared information about the study via their Facebook page and by email to its members. The participants were recruited from November the 20th to November the 27th 2019.

Inclusion criteria for the study were: 1) age ≥ 18 years; 2) a diagnosis of ADHD; 3) impairment in everyday functioning; 4) access to a computer and/or smartphone and the Internet, and 5) speaks, writes, and reads Norwegian.

Exclusion criteria were: 1) ongoing suicidality; 2) ongoing substance abuse; 3) other severe mental disorders, such as bipolar disorder or psychosis; 4) engagement in another psychological treatment program.

### 2.3 Procedure

A local ADHD patient association sent an email to its members with information about the study and a link to an open website with information about the present study. The link to the study website was also shared on their Facebook page and re-shared by other local associations. Those who wanted to participate were asked to complete an anonymous online screening to evaluate their eligibility. The online screening included five questions assessing age, the presence of an ADHD diagnosis, skills in Norwegian language, access to a computer, and availability over the next four weeks. Those who met the inclusion criteria were asked to schedule a phone conversation with a clinical psychologist (ESN). During the phone conversation, the participants were asked to confirm an ADHD diagnosis, symptoms, and everyday functioning, and by whom and when they were diagnosed. Co-existing diagnoses and problems were assessed, such as suicidality using item 10 from the Montgomery and Åsberg Depression Rating Scale (MADRS) (Montgomery and Åsberg, 1979), and depression, psychosis, bipolar disorder and substance-abuse using the MINI international neuropsychiatric interview (MINI) (Sheehan et al., 1998). Participants with self-reported diagnoses of depression and bipolar disorder, participants who had experienced a psychotic episode, and participants scoring above 3 on MADRS item number 10 were excluded. In addition, participants were asked about current psychological treatment and their motivation to participate in the study.

Eligible participants were asked to log into a secure portal, read and sign an informed consent form to participate in the study. Pre-intervention assessment was conducted before the participants accessed the first two modules of the intervention. The third module was accessed one week later. The post-intervention assessment was conducted two weeks after access to the third module. Since the intervention was self-guided, the participants did not receive any therapist support, but a generic text message when a new module was made available and if they remained inactive in the intervention for four days. As the primary objective of the study was to evaluate the feasibility of the intervention, recruiting a small sample to test a shorter version of the full intervention was found to be more ethical in consideration of the participants' time. The current treatment platform will also be used in the main trial, and the modules included in the present study are also representable for the remaining modules in terms of structure and layout.

### 2.4 Intervention

The intervention was developed for adults with ADHD and the content is tailored towards key concerns and challenges related to ADHD. The intervention is based on general principles from GMT, DBT and CBT. These approaches have been included in previous intervention programs for adults with ADHD, with promising results (In de Braek et al., 2017; Philipsen et al., 2007; Weiss et al., 2012). The intervention was developed by an interdisciplinary team of end-users, clinicians, and researchers from the fields of clinical psychology, computer science and human-computer interaction. In development of the intervention we applied the person-based approach (PBA), which is a user-centered approach that informs the researcher in planning, developmental and evaluation phases of the intervention, ensuring that the intervention meets the target groups' needs (Yardley et al., 2015). In accordance with PBA, we examined the literature on psychological interventions for adults with ADHD and held several focus group meetings with adults with ADHD to understand their needs and preferences.

The content of the first three modules of the intervention is shown in Table 1. These three modules mainly focused on breathing and inhibition techniques, while the following four modules will focus on emotion regulation strategies, planning and organization techniques, and self-compassion and acceptance strategies. The modules included two or three coping techniques, and the participants were instructed to continue practicing at least one of those for the rest of the week (See Table 2 for overview of coping techniques). All modules also included case vignettes and lived-experience videos which were based on real-life experiences reported by adults with ADHD. These videos and vignettes served to clarify important training principles and to help participants to make connections between the material presented in the program and their own experiences (Flobak et al., 2021). In addition, participants were encouraged to complete weekly action plans, a daily log tracking time and experiences with practicing the techniques, as well as a daily diary throughout the training period.

**Table 1:** Overview of module content.

Module	Content	Tasks
1: Start	• Introduction to the program. • Introduction to key elements such as diary, goal setting and calendar. • Lived-experience videos.	• Description of symptoms and everyday life. • Goal setting. • Diary.
2: Breathe	• Psychoeducation about breathing as a technique for awareness. • Lived-experience videos and case vignettes. • Three breathing exercises.	• Daily breathing exercises. • Logging. • Diary.
3: Stop	• Psychoeducation about inattention, the autopilot and hyperfocus. • Lived-experience videos and case vignettes. • Two stop exercises.	• Give examples of autopilot mistakes. • Give examples of factors that enhance and hinder focused attention. • Daily stop exercise. • Logging. • Diary.

**Table 2:** Overview of coping techniques.

Module	Framework	Coping technique	Description
1	Introduction	None	
2	DBT / mindfulness	Focused breathing	This technique included inhaling for three second and exhaling for three seconds for approximately two minutes. The aim of the exercise is to regain focus and reduce stress.
		Thoughtful breathing	This technique included focusing one's attention to the breath and noticing when one's attention is drifting. The exercise included a three-minute audio file with a narrator that guided the participants. The participants were also given written instructions if they did not wish to use the audio file.
		Attentive walking	This technique included focusing on one's breath while walking. This was an alternative option for those who might feel restless when sitting down and focusing on one's breath.
3	GMT	Attentive awareness in routine activities	This technique included practicing awareness while doing a routine activity, such as doing the dishes or brushing one's teeth. The aim of the technique is to stop the autopilot and enhance one's presence in everyday situations.
		Stop	This technique included stopping during the day to assess whether one's behavior was in line with one's goals and intentions, with the aim of stopping the autopilot. Participants were told that they could use digital or visual reminders to remember to stop. There was also an audio file that the participants could use.

### 2.5 Feasibility outcome measures

#### 2.5.1 Adherence

The adherence was assessed by dropout rates, number of completed modules, log entries and diary entries, and how often the participants practiced the exercises. Information regarding number of completed modules, log entries and diary entries was available for all participants, as this was registered automatically in the treatment platform.

#### 2.5.2 Treatment credibility and satisfaction

Credibility of the intervention was measured by the third item from the Credibility and Expectancy Scale (CEQ) (Borkovec and Nau, 1972): “How confident would you be in recommending this treatment to a friend who experiences similar problems”. Participants were also asked how satisfied they were with the training program, with response options ranging from: “Not satisfied at all” (1) to “Very satisfied” (6), and how well the treatment responded to their problems, with response options ranging from: “Very badly” (1) to “Very good” (6). Moreover, participants were asked how likely they were to continue to use the techniques from the intervention program, with response options ranging from: “Very unlikely” (1) to “Very likely” (4). In addition, open questions were included at the post-assessment regarding useful aspects, difficult and/or challenging aspects, adverse events, and missing elements in the intervention.

### 2.6 Clinical outcome measures

#### 2.6.1 ADHD symptoms

The Adult ADHD Self-Rating Scale (ASRS) is designed to assess core symptoms of ADHD and includes 18 items rated on a five-point scale from: “Never” (0) to “Very Often” (4) (Kessler et al., 2005). The scale has good psychometric properties with a Cronbach's alpha reported to be 0.88 (Adler et al., 2006). The pre-intervention Cronbach's alpha in the current sample was 0.84. In the present study we will report the total score across the 18 items (0–72 points) and two subscales, one including the nine inattention symptoms (inattention subscale) and one including the nine hyperactivity/impulsivity symptoms (hyperactivity subscale) (each 0–36 points).

#### 2.6.2 Stress

The Perceived Stress Scale (PSS) is designed to assess the level of perceived stress during the past month (Cohen et al., 1983). The scale includes 14 items and responses are rated on a five-point scale from “Never” (0) to “Very often” (4). The PSS scale has demonstrated good psychometric properties with a Cronbach's alpha of 0.75–0.89 (Lee, 2012). The pre-intervention Cronbach's alpha in the current sample was 0.78.

#### 2.6.3 Quality of life

Adult ADHD Quality of Life Measure (AAQoL) is designed to assess quality of life during the past two weeks in adults with ADHD (Brod et al., 2006). AAQoL includes 29 items and responses are rated on a five-point scale from “Not at all/Never” (1) to “Extremely/Very Often” (5). The AAQoL yields a total score and four subscale scores: Life Productivity (eleven items), Psychological Health (six items), Life Outlook (seven items), Relationships (five items). AAQoL has demonstrated good psychometric properties with a Cronbach's alpha of 0.93 (Brod et al., 2015). The pre-intervention Cronbach's alpha in the current sample was 0.84.

#### 2.6.4 Depression

The Patient Health Questionnaire-9 (PHQ-9) is designed to assess the presence and severity of different dimension of depressive symptoms during the past two weeks (Kroenke et al., 2001). The scale includes nine items and responses are rated on a four-point scale from “Not at all” (0) to “Almost every day” (3). The PHQ-9 has shown good psychometric properties with Cronbach's alphas of 0.86 and 0.89 (Kroenke et al., 2001). The pre-intervention Cronbach's alpha in the present study was 0.34.

#### 2.6.5 Anxiety

The General Anxiety Disorder 7-Item Scale (GAD-7) is designed to measure anxiety symptoms during the past two weeks (Spitzer et al., 2006). The scale includes seven items and responses are rated on a four-point scale from: “Not at all” (0) to “Almost every day” (3). GAD-7 has shown good psychometric properties with a Cronbach's alpha of 0.93 (Mills et al., 2014). The pre-intervention Cronbach's alpha in the current sample was 0.68.

### 2.7 Statistical analysis

Descriptive statistics were used to evaluate baseline demographics and feasibility outcomes. Paired sample *t*-tests were used to evaluate within-group clinical effects. Cohen's *d* was used to calculate the within-group effect sizes, defined as the mean difference from pre-treatment to post-treatment on each of the outcome measures divided by the pooled standard deviation. Clinically significant improvement was defined as having a Reliable Change Index (RCI) above 1.96 on the total ASRS, while deterioration was defined as having an RCI score below −1.96 on the total ASRS (Jacobson and Truax, 1992). The RCI was assessed by calculating the individual participant's difference from pre-treatment to post-treatment on the total ASRS divided by the standard error (Martinovich et al., 1996). IBM SPSS version 25 was used to conduct all statistical analyses (IBM Corp, 2017).

### 2.8 Ethics

The study was approved by the Norwegian Regional Committees for Medical and Health Research Ethics, Region West (REK-VEST 2019/257). All participants signed an informed consent form.

## 3 Results

### 3.1 Participants

**Fig. 1:**
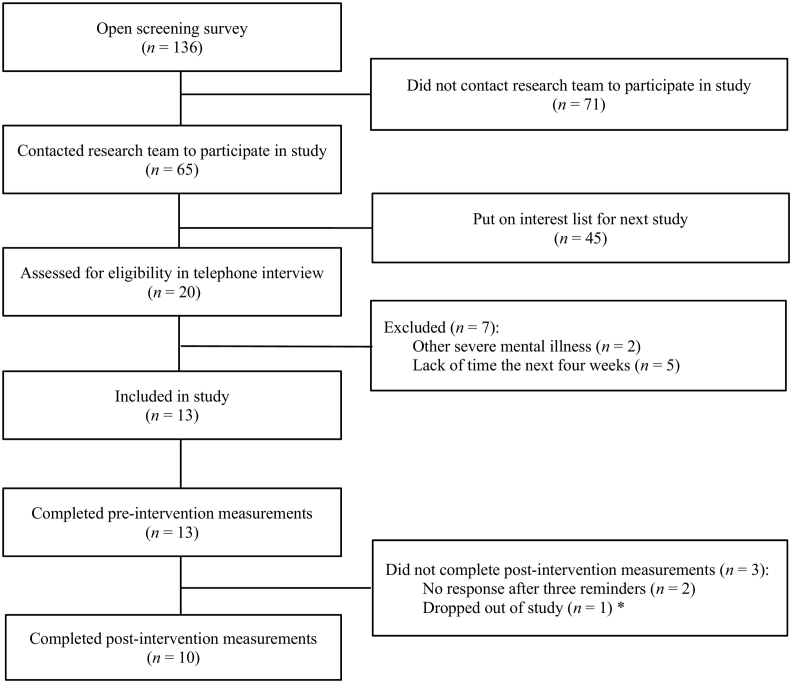
Participant flow chart Note. *One participant contacted the research team shortly after the pre-intervention assessment and gave information that they would not be able to continue the study.

During the recruitment week, 136 individuals completed the online screening survey distributed via the study website (See Fig. 1 for flow chart). Due to constraints in time and healthcare personnel resources, only the first 20 individuals that signed up were assessed in the telephone screening interview.

A total of 13 adults with ADHD were included in the study (See Table 3 for demographics). Ten participants were diagnosed with ADHD in adulthood, where six of them were diagnosed in the past year. None of the participants were engaged in another psychological treatment program, but five participants reported to be involved in non-pharmacological activities related to ADHD, such as attending monthly psychoeducational meetings arranged by the patient association. Three participants also reported to have made changes in their medication during the intervention period. Three participants did not complete the post-intervention assessment, and an additional participant did not complete the ASRS at the post-intervention assessment (See Fig. 1).

**Table 3:** Participant demographics.

	*n* / mean	% / *SD*	Range
Gender			
Female	7	54.8%	
Male	6	46.2%	
Age	39.2	10.2	26–62
Employment			
Full-time employed / student	6	46.2%	
Sick leave / disability pension	7	54.8%	
Education			
Elementary school	2	15.4%	
High school	5	38.5%	
Higher education	6	46.2%	
Use of ADHD medication	11	84.6%	
ASRS full scale	45.8	9.7	31–65

Note. ASRS = Adult ADHD Rating Scale. SD=Standard Deviation.

### 3.2 Feasibility outcomes

#### 3.2.1 Adherence

Mean number of completed modules among all 13 participants was 1.85 (*SD* = 1.3) out of three modules. Seven participants (54%) completed all three modules in the intervention and were thus defined as completers. The seven participants who completed all intervention modules, also completed the post-intervention measurements. Among the non-completers (*n* = 6), one participant never started the program, one participant started the first module, but did not finish, and four participants completed only the first module. Non-completion was reported to be due to illness, stressful life events or busy schedules. Mean number of exercise log entries among all 13 participants was 1.85 (*SD* = 4.4, range = 0–14) and mean number of diary entries was 1.54 (*SD* = 3.3, range = 0–12). Mean self-reported practice time among the ten participants who completed the post-intervention assessment was five days a week (*SD* = 2.4).

#### 3.2.2 Treatment credibility and satisfaction

All ten participants who completed the post-intervention assessment reported that they would recommend the intervention to a friend experiencing similar challenges as themselves. A total of eight participants (80%) reported that they were mostly satisfied, satisfied, or very satisfied with the intervention, with a mean rating = 4.2 (*SD* = 0.9, range = 3–6). The participants reported that the program fitted well to their challenges, with a mean rating = 4.6 (*SD* = 0.8, range = 4–6). They also reported that they expected to continue using the coping techniques, with a mean rating = 3.5 (*SD* = 0.7, range = 2–4).

Seven of the ten participants who completed the post-intervention assessment reported the coping techniques as the most useful component of the intervention. The STOP technique in module three was emphasized by four participants as useful. The logging exercise was most frequently mentioned as a challenge (*n* = 4), both because of its organization and usability, and because it was stressful to complete log entries every day. Other challenges reported were related to technical problems (*n* = 3), such as difficulties when logging into the platform and when saving one's work. Some suggestions for improvements were also reported, such as inclusion of therapist support and an app that would make it easier to access the program. None of the participants who completed the post-intervention assessment (*n* = 10) reported any adverse events, but one participant mentioned feelings of guilt when not being able to complete all the homework assignments.

### 3.3 Clinical outcomes

**Fig. 2:**
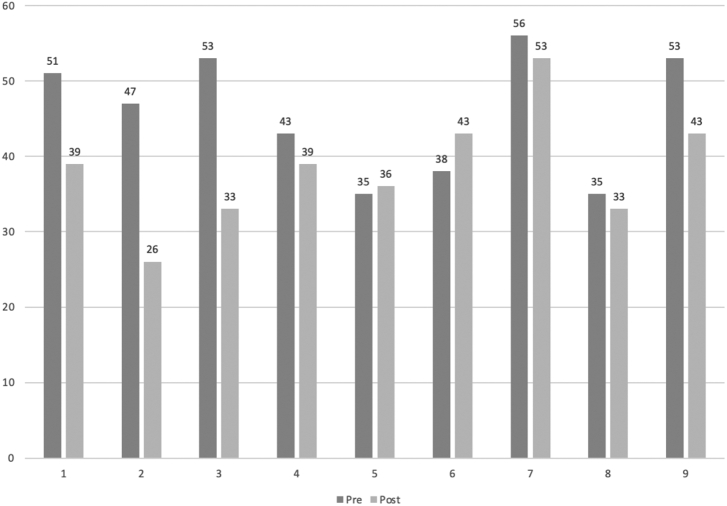
Individual trajectories on the total ASRS score Note. The figure shows the individual scores on the total ASRS among the nine participants who completed the questionnaire at post-assessment.

Within-group effects for all clinical outcome measures are shown in Table 4. The decrease in the total ASRS score from pre to post was statistically significant. The reduction was also significant on the ASRS inattention subscale, while the reduction on the hyperactivity subscale was non-significant. Four participants showed a clinically significant improvement (RCI < 1.96) on the total ASRS, and none of the participants showed a deterioration (RCI > −1.96). The remaining participants (*n* = 5) were defined as non-responders (See Fig. 2. for individual trajectories). Among the completers (*n* = 7), a significant decrease was found for self-reported stress and a significant increase was found for self-reported of quality-of-life. No significant changes were found on measures of anxiety and depression.

**Table 4:** Within-group effects for clinical outcome measures.

Outcome measure	*n*	Pre *M* (*SD*)	Post *M (SD)*	Change (%)	Statical tests	Significance *p*	Effect size Cohens *d*	[95% CI for difference]
ASRS full scale								
All participants^a^	9	45.7 (8.2)	38.3 (7.7)	−16.2%	*t*(8) = 2.429	0.041*	0.93	[+0.4; +14.3]
Completers^b^	6	47.5 (7.6)	38.8 (9.1)	−18.0%	*t*(5) = 2.925	0.033*	1.04	[+1.1; +16.2]
ASRS Inattention								
All participants	9	26.7 (3.5)	21.2 (3.5)	−23.0%	*t*(8) = 3.696	0.006*	1.57	[+2.1; +8.8]
Completers	6	27.7 (3.1)	21.7 (4.2)	−24.3%	*t*(5) = 3.286	0.022*	1.63	[+1.3; +10.7]
ASRS Hyperactivity								
All participants	9	19.0 (6.3)	17.1 (5.0)	−10.5%	*t*(8) = 1.046	0.326	0.33	[−2.3; +6.1]
Completers	6	19.8 (5.4)	17.2 (5.6)	−14.1%	*t*(5) = 2.039	0.097	0.47	[−0.7; +6.0]
PSS								
All participants	10	31.3 (5.6)	28.8 (8.6)	−8.0%	*t*(9) = 1.100	0.300	0.35	[−2.6; +7.6]
Completers	7	30.6 (5.4)	25.9 (8.4)	−15.0%	*t*(6) = 2.569	0.042*	0.67	[+0.2; +9.2]
PHQ-9								
All participants	10	10.3 (2.6)	9.3 (3.5)	−9.7%	t(9) = 0.791	0.450	0.32	[−1.9; +3.9]
Completers	7	9.7 (2.6)	8.4 (3.9)	−13.0%	t(6) = 0.727	0.495	0.39	[−3.0; +5.6]
GAD-7								
All participants	10	7.2 (2.6)	7.1 (2.7)	−1.4%	t(9) = 0.091	0.930	0.04	[−2.4; +2.6]
Completers	7	7.3 (2.4)	7.0 (3.1)	−4.0%	t(6) = 0.269	0.797	0.11	[−2.3; +2.9]
AAQoL								
All participants	10	48.0 (9.6)	55.8 (15.0)	+16.3%	*t*(9) = −1.822	0.102	0.62	[−17.4; +1.9]
Completers	7	48.4 (11.5)	60.8 (13.5)	+26.0%	*t*(6) = −3.307	0.016*	0.99	[−21.6; −3.2]
AAQoL Relationships								
All participants	10	56.0 (21.1)	64.0 (18.7)	+14.3%	*t*(9) = −1.432	0.186	0.40	[−20.6; +4.6]
Completers	7	53.6 (24.3)	68.6(16.0)	+28.0%	*t*(6) = −2.598	0.041*	0.73	[−29.1; −0.9]
AAQoL Psychological health								
All participants	10	48.3 (15.9)	56.3 (15.7)	+16.6%	*t*(9) = −1.039	0.326	0.51	[−25.2; +9.3]
Completers	7	41.7 (12.3)	60.1 (14.6)	+44.1%	*t*(6) = −4.155	0.006*	1.36	[−29.3; −7.6]
AAQoL Life outlook								
All participants	10	45.0 (10.1)	51.4 (20.2)	+14.2%	*t*(9) = −1.561	0.153	0.40	[−15.7; +2.9]
Completers	7	49.0 (9.4)	59.2 (18.8)	+20.8%	*t*(6) = −2.059	0.085	0.69	[−22.3; +1.9]
AAQoL Life productivity								
All participants	10	46.1 (14.2)	54.6 (16.9)	+18.4%	*t*(9) = −2.054	0.070	0.55	[−17.7; +0.9]
Completers	7	49.4 (15.5)	58.8 (17.7)	+19.0%	*t*(6) = −2.054	0.086	0.57	[−20.6; +1.8]

*Note*. *M* = mean; *SD* = standard deviation. ASRS = Adult ADHD Self-Report Scale; PSS = Perceived Stress Scale; PHQ = Patient Health Questionnaire; GAD = General Anxiety Disorder; AAQOL = Adult ADHD Quality of Life.

a All participants: All participants who completed the post-intervention measurements.

b Completers: Participants who completed all three modules in the intervention.

## 4 Discussion

The aim of the current study was to investigate the feasibility (i.e., adherence, credibility, and treatment satisfaction) of a self-guided Internet-delivered intervention for adults with ADHD. Seven out of thirteen included participants completed all intervention modules, indicating that the adherence to intervention was relatively low. The participants in the study reported both good satisfaction and credibility of the Internet-delivered intervention. Although the study was uncontrolled and the sample size was small, the participants in the study did report a reduction in self-reported symptoms of inattention. Moreover, the participant who completed all intervention modules also reported a reduction in levels of stress and increase in reports of quality-of-life. Thus, the credibility, satisfaction and preliminary short-term indication of efficacy suggest that it is feasible to carry out a larger trial examining the clinical effects of the intervention. However, the adherence to the intervention may indicate that the current intervention is not optimal for adults with ADHD. Moreover, the uncontrolled design and the small sample size of the study limits the robustness of the findings.

### 4.1 Adherence

Adherence was assessed by dropout rates, number of completed modules and exercises. The results showed that adherence among the participants in this trial was relatively low, which is a cause for concern for the main study. Of the thirteen participants who initially were included in the study, six participants (46%) did not complete all intervention modules and several participants did not complete the daily logging and diary exercises. However, other studies of self-guided Internet-delivered interventions for adults with ADHD have reported dropout rates of 77% (Pettersson et al., 2017). Moreover, a meta-analysis on self-guided interventions for depression found that almost 60% of participants drop out before completing 50% of the intervention modules (Karyotaki et al., 2015). As such, the adherence to the current intervention is not lower than expected based on previous studies. Still, given the low number of participants and the design of the current study, the results must be interpreted as preliminary.

Participants explained their non-completion by factors like illness, stressful life events or busy schedules. The lack of sustained adherence may also have other causes. The intervention did not include therapist support and it was not individually tailored, two limitations that are associated with dropout in Internet-delivered interventions (Fernández-Álvarez et al., 2017). The lack of sustained adherence may also be related to characteristics associated with ADHD, such as procrastination, distractibility and forgetfulness, which may challenge commitment to an intervention (National Institute for Health and Care Excellence, 2019). Taken together, these findings emphasize the importance of improving adherence in future studies. This may be obtained by improving the usability of the platform (Eysenbach, 2005), including more event-based or frequent reminders (Horsch et al., 2017), by providing rewards or motivators (Christensen et al., 2009), or by giving the participants more time to complete the intervention modules (Moëll et al., 2015). Moreover, in the future we will aim to improve adherence by tailoring the intervention and by allowing the participants to complete the intervention in a non-linear fashion, where they choose the modules and the order of the modules themselves. It is also likely that therapist support would have been beneficial for treatment adherence (Pettersson et al., 2017). However, it should be emphasized that self-guided interventions have certain advantages, such as cost-effectiveness and increased accessibility to treatment in the population (Powell et al., 2020). As such, smaller effects may still be of significance in a public health perspective (Karyotaki et al., 2017). Moreover, although there might be ways to increase adherence and improve the current intervention, the self-guided Internet-delivered format will likely not be suitable for all. Hence, it will be of importance to further investigate who benefits from this format.

### 4.2 Treatment credibility and satisfaction

Treatment credibility and satisfaction was assessed by self-report questionnaires. The participants' reports of treatment satisfaction are in accordance with findings from previous studies on Internet-delivered interventions for adults with ADHD (Moëll et al., 2015). Although the intervention obtained an overall good rating, the participants also reported shortcomings and recommended improvements, such as technical issues and low usability. In sum, these findings highlight that certain aspects of the intervention should be improved before conducting the main trial. The current study was a feasibility study that only investigated the first three modules of a seven-module intervention and therefore the content of the intervention will be more extensive in the main trial. With the present study, we confirmed that the participants understood the modules and that they considered the content of the modules as relevant to their daily-life challenges. By this, the findings were of great importance to our work to finalize the seven-module intervention program for a randomized-controlled trial.

### 4.3 Preliminary findings on short-term clinical effects

Preliminary clinical outcomes were assessed by self-reported measures of inattention, hyperactivity, depression, anxiety and stress, and quality of life.

#### 4.3.1 ADHD symptoms

The participants reported a significant reduction in the severity level of inattention following the intervention. This is in accordance with results from previous studies on Internet-delivered interventions for adults with ADHD (Moëll et al., 2015; Pettersson et al., 2017). However, the current results must be interpreted with caution due to the small sample size and the uncontrolled design. Moreover, we did not find any significant changes on the ASRS hyperactivity subscale, which may be explained by the intervention's primary focus on inattention or by a floor-effect due to the relative low score on the hyperactivity subscale reported at baseline.

Four of the nine participants who completed the ASRS at post-assessment (44%) showed a clinically significant improvement on the total ASRS. This result is comparable to the clinically significant change in 21% of the adults with ADHD reported by Moëll et al. (2015) since they used a stricter criterion for clinically significant change than the present study. A larger improvement in ADHD symptoms may not be expected, considering that the diagnostic category in adults is defined by the persistence of these symptoms (American Psychiatric Association, 2013). Furthermore, the heterogeneity characterizing the group of adults with ADHD makes it challenging to meet individual needs by one intervention program (Luo et al., 2019).

#### 4.3.2 Stress, quality of life, anxiety, and depression

A significant reduction in stress and a significant increase in quality of life were reported by the seven participants who completed all three modules of the intervention, leaving anxiety and depression unchanged. Interestingly, the quality-of-life score showed the largest improvement from baseline to the post-intervention assessment among the completers. Due to the chronic and persistent nature of ADHD, a growth of positive qualities is likely as important as a reduction in ADHD symptoms, expecting to contribute to a milder impairment in everyday life situations. These findings may indicate that measures of positive qualities can be valuable to include as an outcome measures in psychological intervention studies for adults with ADHD.

## 5 Limitations

The current study has several limitations. As it was a feasibility trial, we found it acceptable to include a small sample size and to use an uncontrolled design. Yet, the small sample size makes all the statical tests severely underpowered and the results must therefore be interpreted with caution. Moreover, three participants did not complete the post-intervention assessment, and there is a possibility that they were less satisfied than the ten participants who completed the post-assessment. In addition, most of the participants used medication for their ADHD, and three participants made changes to their medication during the trial period, making it difficult to disentangle the effects of the intervention from the effects of the medication. However, the purpose of the intervention is not to replace medication, but rather to be used a supplement to pharmacological treatment. Another limitation is the low Cronbach's alpha (0.34) of the PHQ-9. This may indicate that the PHQ-9 was not well-suited for measuring depressive symptoms in the current sample.

The lack of formal confirmation of the ADHD diagnosis may also be considered as a limitation, as we only asked participants to recall the date, venue, and the clinician in charge of the diagnosis. However, the aim of the full trial is to make the intervention available to a nation-wide group of people with ADHD related challenges in their every-day life. We therefore asked for self-reported ADHD diagnosis rather than conducting a full clinical assessment of each participant. Although we are aware of biases related to self-reports, we found it reassuring that all participants met the cut-off score of 17 points on one of the scales assessing current ADHD symptoms (ASRS), and that all participants reported well-known challenges related to the ADHD symptoms in everyday life. Finally, all participants contacted the research team to sign up for the study and we excluded those with other mental illnesses, making the results most valid for well-functioning adults with ADHD.

## 6 Implications and future directions

Overall, the study contributed with information on necessary adjustments and improvements before running a randomized-controlled trial examining the clinical effects of a full intervention with seven modules. The relative low adherence in the current study, underscores the importance of including methods to increase adherence and engagement in self-guided Internet-delivered interventions for adults with ADHD. From the results we suggest that solid work should be put into the usability of the digital platform. Finally, controlled trials with larger sample sizes are called for to obtain results that are valid for the heterogeneous group of adults with an ADHD diagnosis.

## 7 Conclusions

The current study investigated the feasibility of a self-guided Internet-delivered intervention for adults with ADHD. The participants who completed the study reported both good treatment satisfaction and credibility, but the lack of sustained adherence calls for inclusion of methods to increase engagement. It will also be important to examine characteristics of responders and non-responders in future studies to get a clearer picture of who is suited for this format. Overall, the study gave important information on feasibility and necessary augmentations to the intervention before the main trial.

## Funding

This publication is part of the INTROducing Mental health through Adaptive Technology (INTROMAT) project, funded by The Research Council of Norway (agreement 259293). The funding source had no role in the design, data collection, data analysis, interpretation of data, writing the report or in the decision to submit the article for publication.

## Authors' contributions

ESN contributed to the intervention content, data collection, data analyses, interpretation of results and drafting the manuscript. RMFK was responsible for the study design and intervention content and contributed to the drafting of the manuscript. AJL contributed substantially as the domain expert in the ADHD case, to the intervention content, study design, interpreting results and drafting of the manuscript. TN contributed substantially as the head of INTROMAT, to the study design, data collection, interpretations of results and drafting of the manuscript. All authors read and approved the final version of the publication.

## Declarations of competing interest

The authors declare that they have no competing interests.
